# Diagnosing facial synkinesis using artificial intelligence to advance facial palsy care

**DOI:** 10.1038/s41598-025-08548-4

**Published:** 2025-07-09

**Authors:** Leonard Knoedler, Christian Festbaum, Jillian Dean, Helena Baecher, Grégoire de Lambertye, Maximilian Maul, Thomas Schaschinger, Tobias Niederegger, Alexandra Scheiflinger, Michael Alfertshofer, Khalil Sherwani, Claudius Steffen, Max Heiland, Steffen Koerdt, Samuel Knoedler, Andreas Kehrer

**Affiliations:** 1 Department of Oral and Maxillofacial Surgery, Corporate Member of Freie Universität Berlin, Humboldt-Universität zu Berlin, Charité – Universitätsmedizin Berlin, Augustenburger Platz 1, Berlin, Germany; 2 Department of Plastic, Hand and Reconstructive Surgery, Hospital Ingolstadt, Ingolstadt, Germany; 3 School of Medicine, University of Pittsburgh, Pittsburgh, PA USA; 4 Department of Oral and Maxillofacial Surgery, University Hospital Regensburg, Regensburg, Germany; 5 Department of Informatics, Vienna Technical University, Vienna, Austria; 6 Department of Informatics, National Institute of Applied Sciences of Lyon (INSA), Villeurbanne, France; 7 Department of Surgery, Beth Israel Deaconess Medical Center, Harvard Medical School, Boston, MA USA; 8 Department of Plastic, Hand and Reconstructive Surgery, University Hospital Regensburg, Regensburg, Germany; 9 Medical University of Vienna, Vienna, Austria

**Keywords:** Synkinesis, Artificial intelligence, AI, Facial palsy, Facial paralysis, Convolutional neural network, Diagnosis, Medical imaging

## Abstract

Facial palsy (FP) can lead to significant psychological and physical burdens such as facial synkinesis. This involuntary simultaneous movement of facial musculature remains challenging to diagnose and treat. This study aimed to develop a cost-effective, rapid, and accurate artificial intelligence (AI)-based algorithm to screen FP patients for facial synkinesis. Data from 70 FP patients were collected at the University Hospital Regensburg and compared to healthy controls from an online platform. The standardized patient image series included 9 images, of which 3 were used to train the algorithm. The control images were single images. A total of 385 images were used to train and evaluate a convolutional neural network (CNN). The dataset was divided into training (285 images), validation (29 images), and test sets (71 images). The model was trained over 18 epochs. A web application was developed for practical use. The model achieved an accuracy of 98.6% on the test set, correctly identifying 31 of 32 synkinesis cases and all 39 images of healthy individuals. Performance metrics included an F1-score of 98.4%, precision of 100%, and recall of 96.9%. The web application allowed for image upload and rapid synkinesis prediction. The CNN-based model demonstrated high accuracy in detecting synkinesis in FP patients, offering potential for improved diagnostic accuracy and expedited treatment. Further validation with larger datasets is necessary to ensure robustness and generalizability.

**Supplementary Information:**

The online version contains supplementary material available at 10.1038/s41598-025-08548-4.

## Introduction

Facial palsy (FP) represents a debilitating condition that affects up to 2,000,000 people worldwide per year^1^. FP has been associated with significant psychological and physical burdens, as well as a poor quality of life^2,3^. The majority of patients, approximately 70% of cases, are diagnosed with idiopathic FP (Bell’s palsy), followed by trauma and viral infection^4,5^.

Among a wide array of possible FP symptoms, facial synkinesis remains a persistent challenge for both patients and providers^6^. Facial synkinesis is defined as the pathological, involuntary, and simultaneous movement of multiple facial muscles^7^. Such synkinetic movements can affect different facial expressions and may worsen over time^8^. While previous research has suggested that aberrant regrowth of re-sprouting axons may trigger facial synkinesis, future research is warranted to thoroughly decipher the pathogenesis of facial synkinesis^9^.

Recent research has proposed different conservative and surgical approaches for managing facial synkinesis. For example, Azizzadeh et al. have popularized the concept of selective neurectomy, which involves identifying and dissecting the respective nerve branches^9^. Further, patients may benefit from Botulinum toxin injections as well as neuromuscular retraining, selective myectomy, and reanimation procedures^10,11^. However, rapid and reliable diagnosis is paramount for triaging patients to the appropriate treatment pathway^12^.

Currently, the diagnosis of facial synkinesis is primarily based on clinical experience, with a paucity of objective and readily available diagnostic tools^13–15^. Over the past decades, various computer-aided diagnostic programs have shown promising performance but oftentimes require expert knowledge and experience in treating FP patients^16^. Therefore, diagnosis-making can be challenging for non-FP providers (e.g., general practitioners), leading to significant treatment delays^3^. Our group recently developed an effective and reliable automated artificial intelligence (AI)-based algorithm to detect lagophthalmos in FP patients^17^.

In this study, we aimed to program and test a cost-effective, rapid, and accurate algorithm to screen FP patients for facial synkinesis. Our algorithm may assist non-FP providers in effectively diagnosing FP and referring FP patients to FP specialists for further treatment. Ultimately, this line of research may facilitate future cross-disciplinary efforts and improve the diagnosis and treatment of FP patients.

## Methods

From June 2020 to May 2021, prospective data acquisition was performed on 70 patients seen at the Department of Plastic, Hand, and Reconstructive Surgery at the University Hospital Regensburg, Germany. The study was approved by the IRB of the University of Regensburg (No. 20-2081-101). In addition, comparative data were gathered from healthy patients as the control group. All patient images were taken in one dedicated hospital room at the same spot to ensure standardized data collection^18,19^. All study subjects provided informed consent for publication of identifying information/images in an online open-access publication. The standardized patient image series included 9 images, of which 3 were used to train the algorithm. The control images were single images. Overall, we obtained 385 patient images, which were used to train and evaluate a convolutional neural network (CNN). For healthy controls, we accessed a free-to-use online image library (https://de.freepik.com/). A total of 30 healthy controls were included.

The dataset was divided into training, validation, and test subsets, ensuring that images from the same patient were confined to a single subset. This approach was adopted to prevent data leakage and to ensure that the model’s performance metrics were not artificially inflated by having multiple images of the same patient across different subsets. Each image in the dataset was preprocessed to standardize the format and quality. The preprocessing steps included 2 steps: (i) cropping: All images were cropped to a square format to maintain consistency in the input dimensions (ii) resizing: The cropped images were then resized to 256 × 256 pixels to reduce computational load and memory usage. These preprocessing steps were essential to ensure that the input data was uniform and manageable, facilitating efficient training and evaluation of the model.

**Fig. 1:**
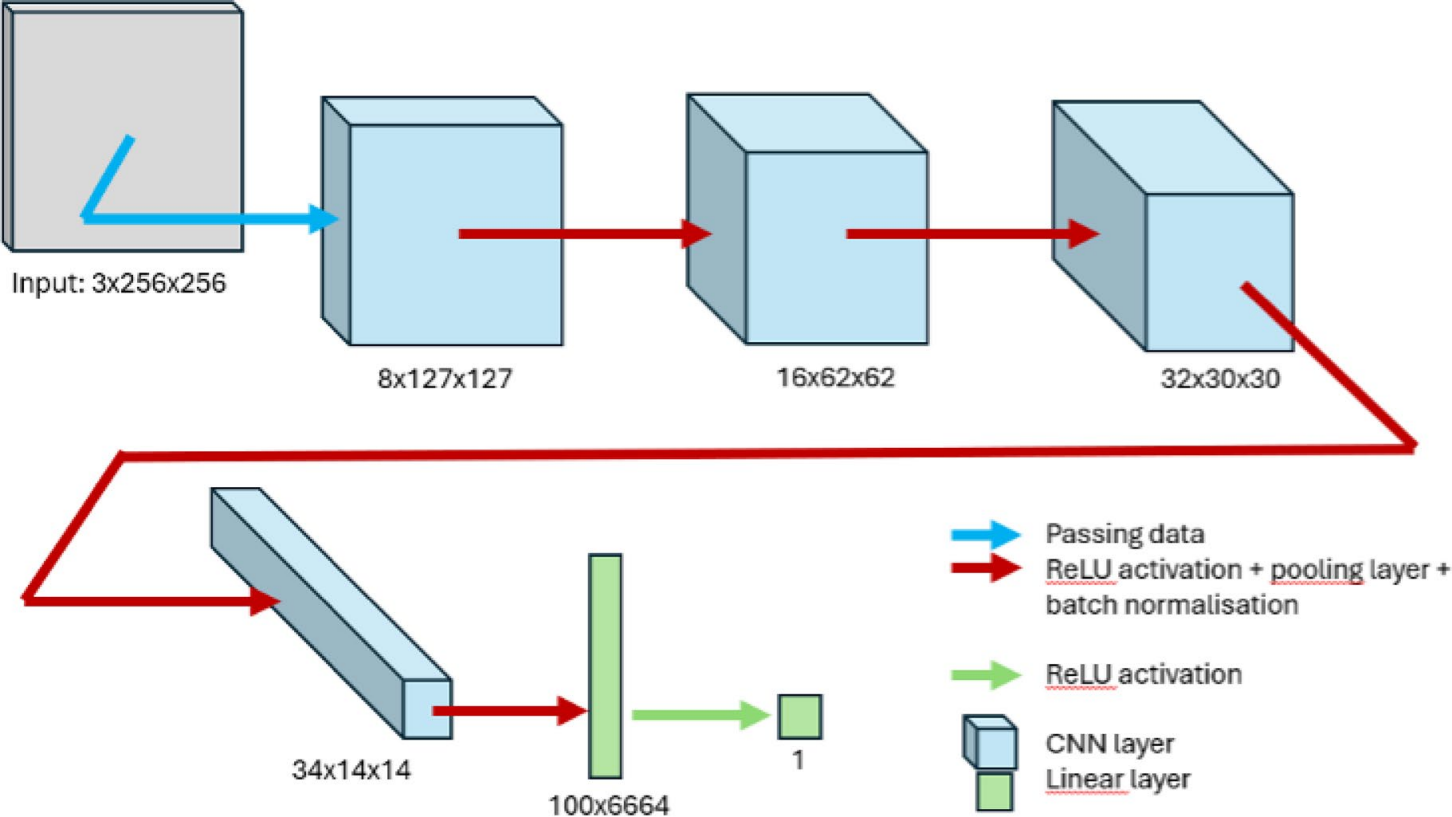
Model architecture diagram. This figure outlines the architecture of the CNN used in the study for diagnosing facial synkinesis. The model consists of several layers starting from the input layer (3 × 256 × 256), progressing through multiple convolutional layers with ReLU activations, pooling layers, and batch normalization, and concluding with a linear layer that outputs the final prediction.

**Fig. 2:**
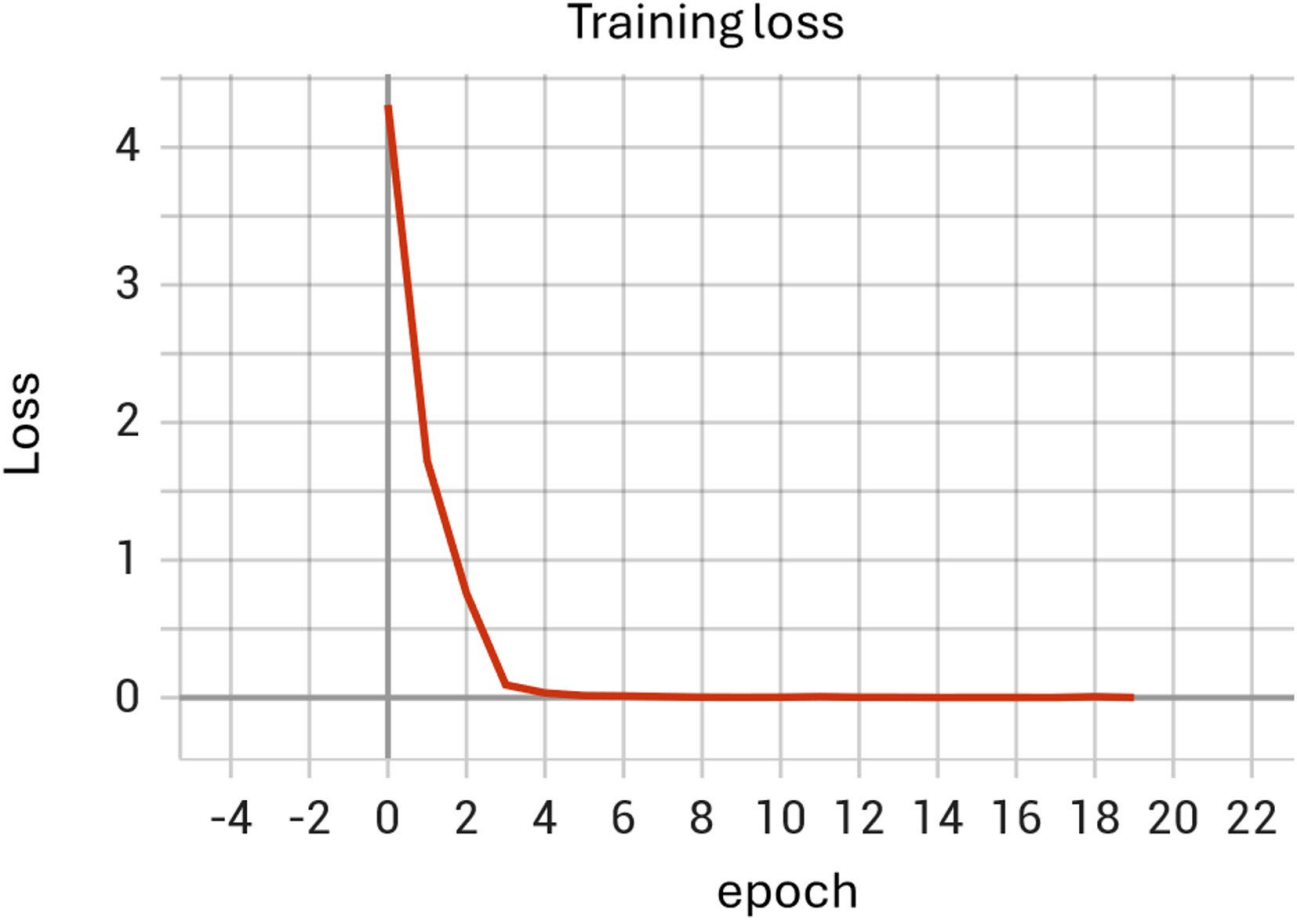
Training loss plot. This plot depicts the training loss of our convolutional neural network (CNN) model over 18 epochs, demonstrating the model’s learning progression. The rapid decline in loss at the initial epochs indicates effective learning, with the loss stabilizing at a low level, which suggests that the model efficiently minimized the error in predicting synkinesis among facial palsy patients during the training phase.

**Fig. 3:**
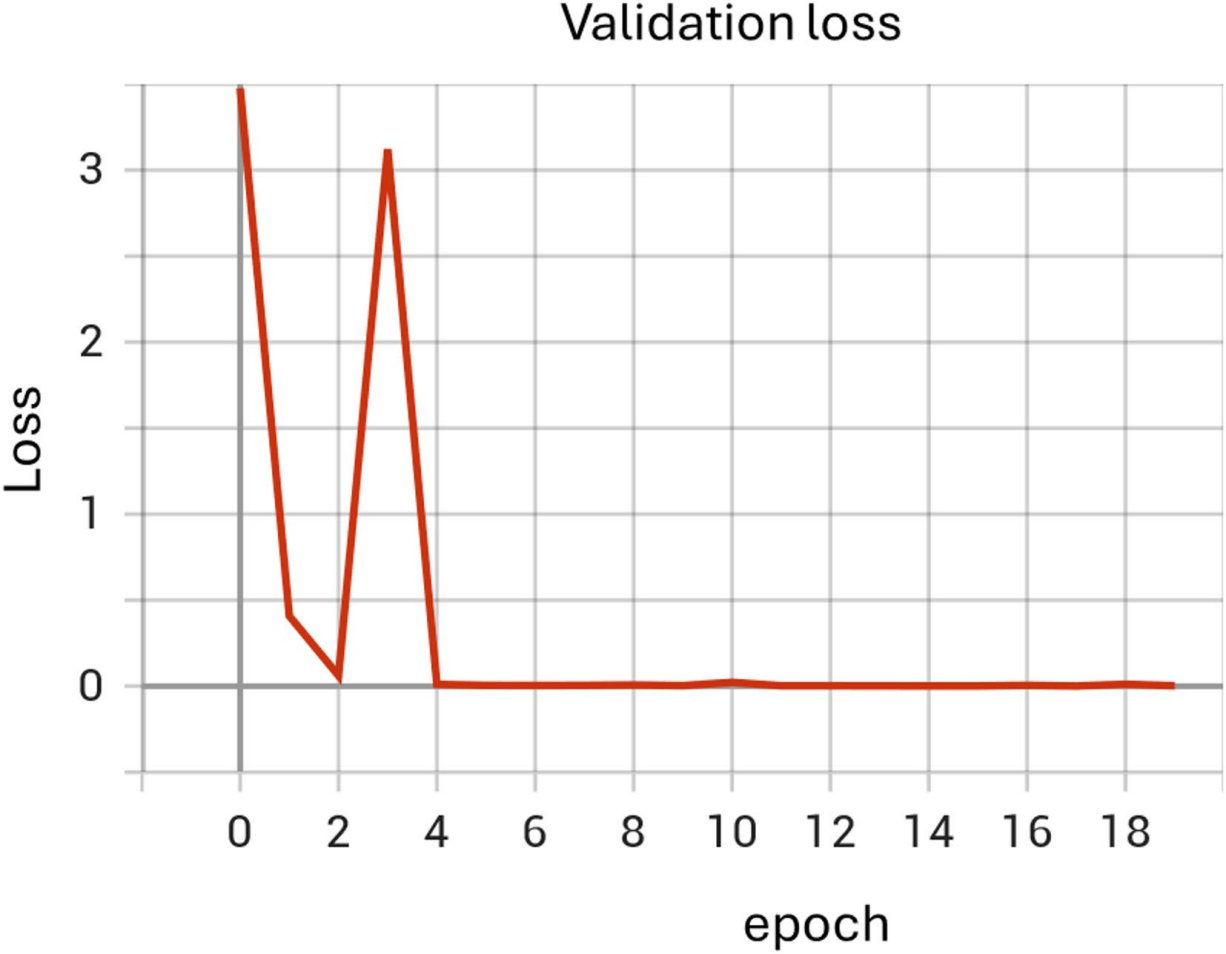
Validation loss plot. This plot shows the validation loss across the same 18 epochs. Notable fluctuations in the loss suggest moments of learning adjustment, which is typical when a model encounters new or complex data patterns in the validation set. The rapid decline followed by stability in loss indicates that the model effectively generalized its predictions to new data, maintaining robustness against overfitting.

A classic computer vision model combining convolutional neural network (CNN) layers, pooling layers, and linear layers was designed to classify images of individuals as either healthy or having facial palsy (specifically synkinesis). The model’s architecture is illustrated in the model structure diagram (Fig. 1) and the code available on github^1^. The model was trained over 18 epochs to optimize its performance. The dataset was divided into three subsets: a training set composed of 285 images (132 healthy and 153 patients with facial palsy), a validation set consisting of 29 images (20 healthy cases and 9 images of FP patients), and a test set comprising 71 images (39 healthy and 32 patients with facial palsy). (Figures 2 and 3)

The distinction was made between healthy individuals and those with synkinesis in repose, during full-effort smile, and forced eye closure. After the training phase, the model achieved: accuracy [%]; recall (i.e., sensitivity) [%]; precision (i.e., the accuracy of the positive predictions) [%]; F1-score (i.e., the mean of precision and recall) [%]; image processing time [ms]; total development costs [US Dollar (USD)]. Additionally, a lightweight (i.e., an application design that is minimalistic, efficient, and quick to respond) web application was developed to facilitate practical application ( https://github.com/GregoireLamb/synkinesis_classifier). (Supplementary Video 1)

## Results

### Model performance on test set

The performance of the model was rigorously evaluated using a test set comprising 71 previously unseen images. The test dataset included 39 images of healthy individuals and 32 images of patients with synkinesis. The model demonstrated a high overall accuracy of 98.6%, with only one instance where a patient with synkinesis was misclassified as healthy.

### Accuracy and performance metrics

The model correctly identified 31 out of 32 pictures of patients with synkinesis and all 39 healthy individuals. This high level of accuracy is reflected in the following performance metrics: an F1-score of 98.4%. The precision was 100%, signifying that every individual identified as having synkinesis indeed had the condition. The recall was 96.9%, indicating that the model successfully detected nearly all patients with synkinesis.

### Confusion matrix analysis

**Fig. 4:**
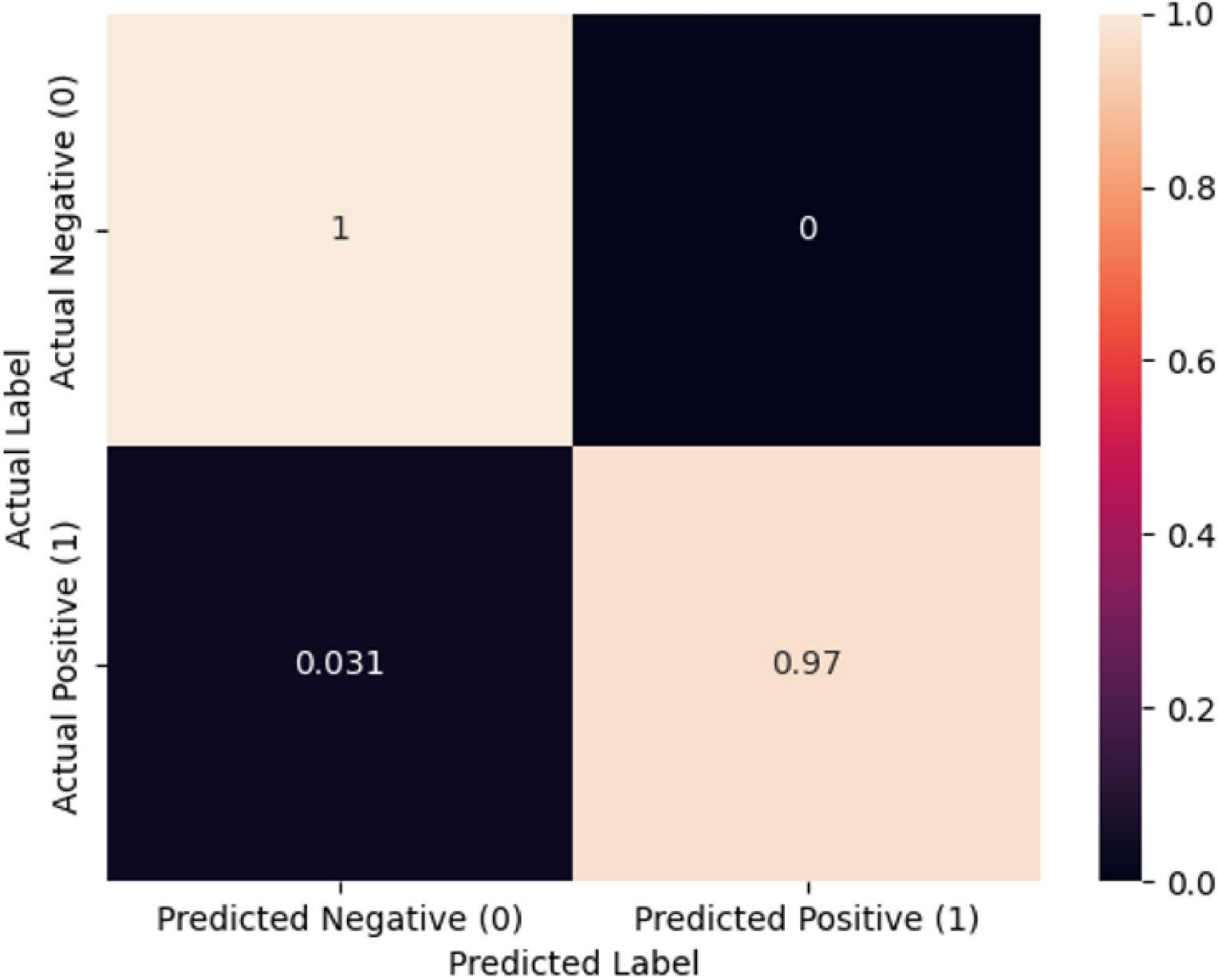
Confusion matrix. This confusion matrix displays the performance of the convolutional neural network (CNN) model in distinguishing between patients with facial synkinesis and healthy individuals. The matrix illustrates the counts of true positives (0.97), true negatives (1), false positives (0.031), and false negatives (0) as fractions of the total test set. Such a matrix helps in visualizing the accuracy, recall, precision, and F1-score of the diagnostic model.

The detailed confusion matrix for the test set further illustrates the model’s robustness. It showed 36 true negatives, meaning healthy individuals were correctly identified as healthy. There were 3 false negatives, where patients with synkinesis were incorrectly classified as healthy. Additionally, there were 3 false positives, where healthy individuals were incorrectly identified as having synkinesis, and 29 true positives, where patients with synkinesis were correctly identified. (Fig. 4)

### Implementation of the model and model development costs

The lightweight web application showed high user friendliness and allowed users to drag and drop an image for prediction. Total development costs amounted to $311.

### Model image processing and training times

For the testing set, mean image processing time was 24 ± 11 ms. The overall training time amounted to 14.4 min. Total development costs amounted to $311 USD.

## Discussion

Over the past decade, a plethora of software applications have emerged in the field of patient medical care, supporting the diagnosis and management of various clinical conditions^14,15,20–22^. Our study contributes to this evolving field by introducing a novel application for holistic synkinesis diagnosis and leveraging the power of convolutional neural networks (CNN) to analyze images of periocular regions.

The development and validation of our CNN-based model for diagnosing facial synkinesis in FP patients mark a significant advancement in the realm of automated medical diagnostics. Our model demonstrated a high degree of accuracy (98.6%) in distinguishing between healthy individuals and those with synkinesis, with an F1-score of 98.4%, precision of 100%, and recall of 96.9%. These metrics highlight the model’s robustness and reliability, rendering it a valuable tool for clinicians. The confusion matrix analysis provided further insights into the model’s performance, revealing only one misclassification among the 71 test images. These metrics echo findings from previous work in diagnosing sequelae of FP. For example, our group reported comparable metrics for CNN-based assessment of lagophthalmos. Using a training set of 826 images, the validation accuracy was 97.8% over the span of 64 epochs^17^. Another study leveraged a CNN to automatically identify (peri-)ocular pathologies such as enophthalmos with an accuracy of 98.2%, underscoring the potential of neural networks when diagnosing facial conditions^23^. Such tools can broaden access to FP diagnostics, thus reducing time-to-diagnosis and effectively triaging patients to the appropriate treatment pathway (e.g., conservative therapy, cross-face-nerve-grafts)^2,3,14,24^. Overall, our CNN adds another highly accurate diagnostic tool for reliably detecting facial pathologies, especially in FP patients.

Another strength of our CNN lies in its high user-friendliness and rapid processing and training times. The mean image processing time was 24 ± 11 ms, and the overall training time was 14.4 min. The development of a lightweight, dockerized web application enhanced the model’s practicality and accessibility. In addition, the total development costs of the CNN were only $311 USD. Such parameters have been identified as key parameters for impactful AI research and effective integration into clinical workflows^25–27^. More precisely, the short training times may pave the avenue toward additional AI-supported diagnostic tools in FP care to detect common short- and long-term complications of FP (e.g., ectropion, hemifacial tissue atrophy). The easy-to-use and cost-effective web application may facilitate clinical use for healthcare providers in low- and middle-income countries, where the incidence and prevalence of FP are higher compared to the high-income countries^28^. To facilitate the download and use of our algorithm, we (i) uploaded the code to GitHub (San Francisco, USA), (ii) integrated the code into an application, and (iii) recorded an instructional video that details the different steps. Healthcare providers from low- and middle-income countries only require an internet connection to install the application. The instructional video will then guide them through the next steps to set up the application and start screening patients. Our application is free to use, and the number of daily screens is not limited. The rapid processing times also carry the potential to increase the screening throughput, further broadening the access to FP care and reducing waiting times for FP patients^3^. Collectively, the CNN represents a rapid, user-friendly, and cost-effective tool.

While our study presents promising results, it is not without limitations. The relatively small sample size, especially for the validation and test sets, suggests the need for further validation with larger and more diverse (i.e., multi-center, -racial, -surgeon) datasets to ensure the model’s robustness and generalizability. Additionally, the model’s ability to distinguish synkinesis from other facial conditions was not evaluated in this study, representing an area for future research. Moreover, integrating our model into clinical practice will require careful consideration of various factors, including user training, data privacy, and the ethical implications of automated diagnostics. Ensuring that clinicians are adequately trained to use the model and interpret its results is essential for maximizing its benefits. Additionally, robust data privacy measures must be implemented to protect sensitive patient information, particularly when using web-based applications. Thus, further validation is essential before clinical implementation. In a broader context, there are different AI/machine-learning-powered tools that have shown promising outcomes in pre-clinical studies and small patient samples (face transplantation, facial reanimation, etc.)^29–32^. However, these tools remain to be investigated in larger-scale trials and integrated into standard clinical workup. Thus, cross-disciplinary efforts are needed to bridge the gap from bench to bedside and to fuel translational efforts.

## Conclusion

Our study introduces a novel CNN-based application for comprehensive synkinesis diagnosis, representing the to-date largest effort to automate synkinesis diagnosis using AI technology. The results highlight the model’s promising accuracy in diagnosing synkinesis. This application has the potential to improve clinical workflows, enhance diagnostic accuracy, and broaden access to care. Future research is warranted to further elucidate the strengths and limitations of this CNN in the clinical setting. Continued advancements and validation are necessary to ensure safe and effective integration into routine patient care.

## Electronic supplementary material

Below is the link to the electronic supplementary material.

Supplementary Material 1.

Supplementary Material 2.

## Data Availability

The datasets used and/or analyzed during the current study are available from the first author (Leonard Knoedler; leonard.knoedler@charite.de) on reasonable request.

## References

[1] Rajangam, J. et al. Bell palsy: facts and current research perspectives. *CNS Neurol. Disord Drug Targets*. **23**, 203–214. 10.2174/1871527322666230321120618 (2024). 36959147 10.2174/1871527322666230321120618

[2] Knoedler, L. et al. The rise of facial palsy on social media over the last 5 years. *J. Craniofac. Surg.* **34**, 564–570. 10.1097/scs.0000000000009106 (2023). 36730871 10.1097/SCS.0000000000009106

[3] Knoedler, S. et al. An ACS-NSQIP data analysis of 30-Day outcomes following surgery for bell’s palsy. *J. Craniofac. Surg.*10.1097/SCS.0000000000009739 (9900). 10.1097/SCS.0000000000009739 PMC10841222 37695075

[4] De Diego-Sastre, J. I., Prim-Espada, M. P. & Fernández-García, F. The epidemiology of bell’s palsy. *Rev. Neurol.* **41**, 287–290 (2005). 16138286

[5] Zhang, W. et al. The etiology of bell’s palsy: A review. *J. Neurol.* **267**, 1896–1905. 10.1007/s00415-019-09282-4 (2020). 30923934 10.1007/s00415-019-09282-4 PMC7320932

[6] Guntinas-Lichius, O. et al. Pathogenesis, diagnosis and therapy of facial synkinesis: A systematic review and clinical practice recommendations by the international head and neck scientific group. *Front. Neurol.* **13**, 1019554. 10.3389/fneur.2022.1019554 (2022). 36438936 10.3389/fneur.2022.1019554 PMC9682287

[7] Shokri, T., Patel, S., Ziai, K., Harounian, J. & Lighthall, J. G. Facial synkinesis: A distressing sequela of facial palsy. *Ear Nose Throat J.* **1455613211054627**10.1177/01455613211054627 (2021). 10.1177/01455613211054627 34836457

[8] Shokri, T., Azizzadeh, B. & Ducic, Y. Modern management of facial nerve disorders. *Semin Plast. Surg.* **34**, 277–285. 10.1055/s-0040-1721824 (2020). 33380914 10.1055/s-0040-1721824 PMC7759435

[9] Azizzadeh, B. et al. Modified selective neurectomy for the treatment of post-facial paralysis synkinesis. *Plast. Reconstr. Surg.* **143**, 1483–1496. 10.1097/prs.0000000000005590 (2019). 30807497 10.1097/PRS.0000000000005590

[10] Shikara, M., Bridgham, K., Ludeman, E., Vakharia, K. & Justicz, N. Selective neurectomy for treatment of post-facial paralysis synkinesis: A systematic review. *Facial Plast. Surg.* **39**, 190–200. 10.1055/a-1950-4483 (2023). 36155895 10.1055/a-1950-4483

[11] Azizzadeh, B. & Frisenda, J. L. Surgical management of postparalysis facial palsy and synkinesis. *Otolaryngol. Clin. North. Am.* **51**, 1169–1178. 10.1016/j.otc.2018.07.012 (2018). 30170699 10.1016/j.otc.2018.07.012

[12] Heckmann, J. G., Urban, P. P., Pitz, S., Guntinas-Lichius, O. & Gágyor, I. The diagnosis and treatment of idiopathic facial paresis (Bell’s Palsy). *Dtsch. Arztebl Int.* **116**, 692–702. 10.3238/arztebl.2019.0692 (2019). 31709978 10.3238/arztebl.2019.0692 PMC6865187

[13] Kehrer, A. et al. Using high-resolution ultrasound to assess post-facial paralysis synkinesis-machine settings and technical aspects for facial surgeons. *Diagnostics (Basel)* **12**. 10.3390/diagnostics12071650 (2022). 10.3390/diagnostics12071650 PMC9322000 35885554

[14] Knoedler, L. et al. A Ready-to-Use grading tool for facial palsy examiners-automated grading system in facial palsy patients made easy. *J. Pers. Med.* **12**. 10.3390/jpm12101739 (2022). 10.3390/jpm12101739 PMC9605133 36294878

[15] Knoedler, L. et al. Towards a reliable and rapid automated grading system in facial palsy patients: facial palsy surgery meets computer science. *J. Clin. Med.* **11**. 10.3390/jcm11174998 (2022). 10.3390/jcm11174998 PMC9457271 36078928

[16] Boochoon, K., Mottaghi, A., Aziz, A. & Pepper, J. P. Deep learning for the assessment of facial nerve palsy: Opportunities and challenges. *Facial Plast. Surg.* **39**, 508–511. 10.1055/s-0043-1769805 (2023). 37290452 10.1055/s-0043-1769805

[17] Knoedler, L. et al. Diagnosing lagophthalmos using artificial intelligence. *Sci. Rep.* **13**, 21657. 10.1038/s41598-023-49006-3 (2023). 38066112 10.1038/s41598-023-49006-3 PMC10709577

[18] Schaede, R. A. et al. Video instruction for synchronous video recording of mimic movement of patients with facial palsy. *Laryngorhinootologie* **96**, 844–849. 10.1055/s-0043-101699 (2017). 28470660 10.1055/s-0043-101699

[19] Santosa, K. B., Fattah, A., Gavilán, J., Hadlock, T. A. & Snyder-Warwick, A. K. Photographic standards for patients with facial palsy and recommendations by members of the Sir Charles bell society. *JAMA Facial Plast. Surg.* **19**, 275–281. 10.1001/jamafacial.2016.1883 (2017). 28125753 10.1001/jamafacial.2016.1883 PMC5697767

[20] Knoedler, L. et al. Artificial intelligence-enabled simulation of gluteal augmentation: A helpful tool in preoperative outcome simulation? *J. Plast. Reconstr. Aesthet. Surg.* **80**, 94–101. 10.1016/j.bjps.2023.01.039 (2023). 37001299 10.1016/j.bjps.2023.01.039

[21] Horsch, C. H. et al. Mobile phone-delivered cognitive behavioral therapy for insomnia: A randomized waitlist controlled trial. *J. Med. Internet Res.* **19**, e70. 10.2196/jmir.6524 (2017). 28400355 10.2196/jmir.6524 PMC5405291

[22] Knoedler, S. et al. Turn your vision into reality-AI-powered pre-operative outcome simulation in rhinoplasty surgery. *Aesthetic Plast. Surg.*10.1007/s00266-024-04043-9 (2024). 38777929 10.1007/s00266-024-04043-9 PMC11739225

[23] Schulz, C. B., Clarke, H., Makuloluwe, S., Thomas, P. B. & Kang, S. Automated extraction of clinical measures from videos of oculofacial disorders using machine learning: feasibility, validity and reliability. *Eye (Lond)* 1–7. 10.1038/s41433-023-02424-z (2023). 10.1038/s41433-023-02424-z PMC9891656 36725916

[24] Kehrer, A. et al. Objectifying the role of the depressor anguli oris muscle using high-resolution ultrasound: A prospective study. *Plast. Reconstr. Surg.* **152**, 866–870. 10.1097/prs.0000000000010287 (2023). 36780356 10.1097/PRS.0000000000010287

[25] Koçak, B., Cuocolo, R., dos Santos, D. P., Stanzione, A. & Ugga, L. Must-have qualities of clinical research on artificial intelligence and machine learning. *Balkan Med. J.* **40**, 3–12. 10.4274/balkanmedj.galenos.2022.2022-11-51 (2023). 36578657 10.4274/balkanmedj.galenos.2022.2022-11-51 PMC9874249

[26] Norgeot, B. et al. Minimum information about clinical artificial intelligence modeling: The MI-CLAIM checklist. *Nat. Med.* **26**, 1320–1324. 10.1038/s41591-020-1041-y (2020). 32908275 10.1038/s41591-020-1041-y PMC7538196

[27] Khalifa, M. & Albadawy, M. Artificial intelligence for clinical prediction: Exploring key domains and essential functions. *Comput. Methods Programs Biomed. Update*. **5**, 100148. 10.1016/j.cmpbup.2024.100148 (2024).

[28] Monini, S., Lazzarino, A. I., Iacolucci, C., Buffoni, A. & Barbara, M. Epidemiology of bell’s palsy in an Italian health district: Incidence and case-control study. *Acta Otorhinolaryngol. Ital.* **30**, 198 (2010). 21253285 PMC3008145

[29] Knoedler, S. et al. Fibroblasts–the cellular choreographers of wound healing. *Front. Immunol.* **14**, 1233800 (2023). 37646029 10.3389/fimmu.2023.1233800 PMC10461395

[30] Knoedler, L. et al. Application possibilities of artificial intelligence in facial vascularized composite allotransplantation-a narrative review. *Front. Surg.* **10**, 1266399. 10.3389/fsurg.2023.1266399 (2023). 38026484 10.3389/fsurg.2023.1266399 PMC10646214

[31] Knoedler, L. et al. Objective and automated facial palsy grading and outcome assessment after facial palsy reanimation surgery—a prospective observational study. *J. Stomatol. Oral Maxillofac. Surg.* **102211**10.1016/j.jormas.2024.102211 (2024). 10.1016/j.jormas.2024.102211 39732200

[32] Knoedler, L. et al. Objectifying aesthetic outcomes following face transplantation—the AI research metrics model (CAARISMA ^®^ ARMM). *J. Stomatol. Oral Maxillofac. Surg.* **126**, 102277. 10.1016/j.jormas.2025.102277 (2025). 39947010 10.1016/j.jormas.2025.102277

