# Supplementary material for: Diagnosing facial synkinesis using artificial intelligence to advance facial palsy care
Source: Sci Rep. 2025 Jul 9;15:24686. doi: 10.1038/s41598-025-08548-4 (PMC12241590; doi:10.1038/s41598-025-08548-4)
Supplement: Supplementary file 2 — Supplementary Material 2. [file 41598_2025_8548_MOESM2_ESM.docx]

**Supplementary Material**

**Media Legend**

**Supplementary Video 1:** This video demonstrates the functionality of the web application developed to classify facial synkinesis using our convolutional neural network (CNN) model. It showcases a user uploading an image of a patient, the application processing the image, and then displaying the diagnostic result. The video highlights the application's user-friendly interface, rapid processing time, and how it provides immediate feedback on the presence of synkinesis, illustrating its practical application in clinical settings.
